# *Flammulinavelutipes* Polysaccharides Modulate Gut Microbiota and Alleviate Carbon Tetrachloride-Induced Hepatic Oxidative Injury in Mice

**DOI:** 10.3389/fmicb.2022.847653

**Published:** 2022-03-23

**Authors:** Yingyin Xu, Zhiyuan Zhang, Bo Wang, Xiaolan He, Jie Tang, Weihong Peng, Jie Zhou, Yong Wang

**Affiliations:** ^1^Department of Preservation and Processing, Sichuan Institute of Edible Fungi, Chengdu, China; ^2^National-Local Joint Engineering Laboratory of Breeding and Cultivation of Edible and Medicinal Fungi, Chengdu, China; ^3^Scientific Observing and Experimental Station of Agro-microbial Resource and Utilization in Southwest China, Ministry of Agriculture, Chengdu, China

**Keywords:** *Flammulina velutipes*, acute liver injury, oxidative stress, gut microbiota, metabolites of gut microbiota

## Abstract

A carbon tetrachloride-induced acute liver injury mouse model is used to study the regulation of gut microbiota and hepatoprotective effect of polysaccharides from *Flammulina velutipes* (FVPs). The hepatoprotective effect of the FVPs leads to reduced levels of serum aspartate transaminase (AST), alanine aminotransferase (ALT), triglyceride (TG), total cholesterol (TC), total bile acid (TBA) content, and change in liver histopathology. Their anti-oxidant activity is exhibited by decreased levels of hepatic malonaldehyde (MDA) and protein carbonyl (PC) content and increased catalase (CAT) and superoxide dismutase (SOD) content. The anti-inflammatory ability of the FVPs is reflected in a decrease in pro-inflammatory cytokines (including IL-6, IL-1β, and TNF-α). 16S rRNA sequencing shows that the FVPs change the composition of the gut microbiota. A subsequent metabolomics analysis of the gut bacteria (UHPLC–MS/MS-based) revealed that fatty acid biosynthesis, tryptophan metabolism, and metabolism of xenobiotics by cytochrome P450 play important roles in the hepatoprotective effect. This study provides a potential way to modulate gut microbiota and manage liver diseases using natural products.

## Introduction

Acute liver injury is a type of abnormal liver function that can be induced by certain medicines, alcoholism, viral hepatitis, toxins, and hepatic ischemia reperfusion injury (Zhu et al., 2019). Acute liver injury is a potential factor in liver fibrosis, hepatitis, cirrhosis, and liver cancer and can ultimately lead to terminal liver failure (Liu et al., 2014). Oxidative damage induced by reactive oxygen species (ROS) has been shown to play the main role in the development of acute liver injury (Liu et al., 2018).

Carbon tetrachloride (CCl_4_) can be biotransformed to ROS *via* the hepatic cytochrome P450 system, and so CCl_4_-induced mouse models are extensively used to evaluate the effect of hepatoprotective medicines (Zou et al., 2016). A growing amount of evidence suggests that gut microorganisms hold the balance in the contributions natural products make to hepatoprotection (Meng et al., 2018). Gut dysbiosis increases the permeability of the gut barrier, allowing translocated bacteria and leaked gut-derived products to reach the liver *via* the portal vein. This increases the level of oxidative stress and inflammation in the liver and threatens its healthy function (Meng et al., 2018). Although many hepatoprotective drugs have been developed, the majority of them have adverse effects, such as liver fibrosis and even liver failure (Wang et al., 2019; Zhou et al., 2021). Hence, there is increased demand for effective natural products that can protect the liver and reverse gut dysbiosis to use as alternatives to therapeutic agents.

The edible mushroom *Flammulina velutipes* (also known as enokitake or golden mushroom) is cultivated worldwide for its flavor and nutritional value (Fang et al., 2017). *Flammulina velutipes* polysaccharides (FVPs) exhibit a multitude of pharmacological effects. For example, they have been found to show potentially very promising anti-microbial, anti-oxidant, anti-inflammatory, and immunoregulation effects, etc (Dong et al., 2017; Zhang et al., 2018). More specifically, in the context of acute liver injury, FVPs have been reported to have hepatoprotective effects (Pang et al., 2007; Zhang et al., 2018). However, the hepotaprotective effect of FVPs with microbiological and metabolomic changes in gut have not been explored.

In this study, a CCl_4_-induced acute liver injury mouse model is used to evaluate the hepatoprotective effect of newly-harvested FVPs. The study focuses on the oxidative stress and inflammation levels in the liver and is supported by gut microbiota and fecal metabolomics analyses. The aim is to improve our understanding of the symptomatic relief function of the FVPs with respect to CCl_4_-induced acute liver injury.

## Materials and Methods

### Preparation of the FVPs

*Flammulina velutipes* polysaccharides were harvested from the fruitbodies of *F. velutipes* using hot water (100°C for 6 h). A solid–liquid ratio of 1:20 (w/v) was employed. After centrifugation, the supernatant was added to ethanol (3 volumes of ethanol to 1 volume of supernatant) and the resulting precipitate collected. The Sevage method was used to deproteinate the precipitate. After dialysis, the deproteinated part was applied to diethylaminoethyl cellulose (Sigma, United States) at a flow rate of 3.0 ml/min. The column was then eluted with distilled water, 50 mM, 150 mM, and 1 M NaCl successively at flow rates of 10 ml/min. After assessment of their hepatoprotective effects, the 50 mM NaCl eluted part was gathered and lyophilized as FVPs for further research (Xu et al., 2021b).

The monosaccharide composition of the FVPs was determined *via* liquid chromatography (Agilent 1200, United States). The FVPs were passed through a Shiseido C18 column at room temperature at a flow rate of 1 ml/min. The saccharides were analyzed using the standard method outlined by the National Renewable Energy Laboratory (CO, United States): hydrolysis with dilute sulfuric acid together with calibration using standard solutions of xylose, rhamnose, fucose, mannose, arabinose, galactose, galacturonic acid, glucose, glucuronic acid, and ribose (Xu et al., 2021a). The weight-average (Mw) and number-average (Mn) molecular weights of the FVPs were determined *via* high-performance gel permeation chromatography, as previously described in the literature (Xu et al., 2021a). Fourier transform infrared (FT-IR) spectra of the FVPs were captured using an FT-IR microscope (model iN 10, Thermo Nicolet Corp.; Madison, WI, United States) fitted with a liquid nitrogen-cooled mercury-cadmium-telluride detector (Xu et al., 2021b).

### Liver Injury Model and Treatment

Male C57BL/6 mice (32 in number, aged 6–7 weeks with an average weight of 17.03 ± 0.25 g) were purchased from Chengdu Dssy Experimental Animals Co., Ltd (Chengdu, China). They were housed in a specific pathogen-free environment (25°C; cycles of 12 h light followed by 12 h dark) with *ad libitum* access to food and water.

The mice were randomly assigned to one of four groups (*n* = 8 per group): (1) Normal group (with standard diet); (2) Control group (CCl_4_ with standard diet); (3) LFVPs group (CCl_4_ with standard diet plus 100 mg/kg FVPs); and (4) HFVPs group (CCl_4_ with standard diet plus 200 mg/kg FVPs). The experiment lasted 2 weeks. Mice were gavaged with FVPs or normal saline once daily for 14 days. Shortly after the last treatment (2 h), the mice in the Control, LFVPs, and HFVPs groups were injected intraperitoneally with CCl_4_ (0.2% CCl_4_/olive oil mixture) at a rate of 5 ml/kg bodyweight (Zhu et al., 2019). The mice in the Normal group were injected intraperitoneally with the same dose of olive oil. On day 15, the mice were sacrificed *via* anesthesia to yield specimens for subsequent analysis.

### Biochemical Assays

Blood samples were harvested from the coeliac arteries of the mice. The blood was centrifuged and the serum analyzed to determine its aspartate transaminase (AST), alanine aminotransferase (ALT), total cholesterol (TC), triglyceride (TG), and total bile acid (TBA) content (Yang et al., 2015).

Hepatic tissues were collected, homogenized in saline, and centrifuged. The supernatant was then analyzed spectrophotometrically for superoxide dismutase (SOD), catalase (CAT), IL-1β, IL-6, TNF-α, myeloperoxidase (MPO), malonaldehyde (MDA), and protein carbonyl (PC). All the commercial assay kits employed were obtained from the Nanjing Jiancheng Bioengineering Institute (Nanjing, China; Lykkesfeldt, 2007).

### Histology

Fresh liver tissues were cut into 5 mm pieces and fixed in 10% neutral buffered formalin at room temperature for 24 h. They were then dehydrated using a graded series of ethanol solutions before being embedded in paraffin. The tissues were cut into 5–6 μm sections and stained with hematoxylin–eosin (HE). They were then observed using a microscope (Leica Microsystems, Wetzlar, Germany) at magnifications of 100× and 400× (Sun et al., 2017).

### Gut Microbiota Analysis

Samples of the mice’s intestinal contents were collected from colon immediately after sacrifice. The cetyltrimethylammonium bromide/sodium dodecyl sulfate extraction method was employed to obtain the total DNA from the intestinal content. The extracted DNA was subjected to 16S amplification using primers designed to incorporate both the Illumina adapters and a sample barcode sequence, allowing directional sequencing that covers the variable region V4 [primers: 515 F (GTGCCAGCMGCCGCGGTAA) and 806 R (GGACTACHVGGGTWTCTAAT)]. Phusion® High-Fidelity PCR Master Mix (New England Biolabs, United States) was used for the PCR reactions (Lee et al., 2018).

Sequencing libraries were produced using an Ion Plus Fragment Library Kit 48 rxns (Thermo Scientific, United States) according to the manufacturer’s recommendations. Libraries were sequenced on an Ion S5TM XL platform and 400/600 bp single-end reads were generated. The data were based on sequenced reads and operational taxonomic units (OTUs). UPARSE software (v7.0.1001) was used to carry out the analysis. Sequences that have similarities ≥97% are regarded as the same OTUs. The Silva database^1^ was employed to annotate the taxonomic information based on the Mothur algorithm (Xu et al., 2021b).

### Measurement of Fecal Metabolomics

After the intestinal contents had been harvested from colon, each specimen was disposed with liquid nitrogen. The homogenate was resuspended with pre-chilled 80% methanol and 0.1% formic acid, respectively. After centrifugation, the supernatant was diluted with LC–MS grade water until the concentration of the methanol was 60%. The samples were then filtered (0.22 μm filter) and then injected into the LC–MS/MS system for analysis.

LC–MS/MS was performed using a Vanquish UHPLC system (Thermo Fisher) coupled with an Orbitrap Q ExactiveTM series mass spectrometer (Thermo Fisher). The samples were processed using a Hypersil Gold column (100 mm × 2.1 mm, 1.9 μm) at a flow rate of 0.2 ml/min in a 16-min linear gradient. The positive polarity mode consisted of eluent A (0.1% FA in water) and eluent B (methanol). The negative polarity mode consisted of eluent A (5 mM ammonium acetate, pH 9.0) and eluent B (methanol). The solvent gradient applied was as follows: 2% B, 1.5 min; 2–100% B, 12.0 min; 100% B, 14.0 min; 100–2% B, 14.1 min; 2% B, 16 min. The mass spectrometer was used in positive/negative polarity mode using a spray voltage of 3.2 kV, capillary temperature of 320°C, sheath gas flow rate of 35 arb, and auxiliary gas flow rate of 10 arb.

The LIPID MAPS® structure database,^2^ Human Metabolome database,^3^ and Kyoto Encyclopedia of Genes and Genomes (KEGG) database^4^ were employed to annotate the metabolites. Partial least squares discriminant analysis (PLS-DA) was carried out using MetaX. Univariate analyses (*t*-tests) were carried out to determine the levels of statistical significance (*p*-values). Metabolites with VIP scores > 1, value of *p* < 0.05, and fold-changes ≥2 or ≤0.5 were considered to be significantly different (Xu et al., 2021b).

### Data Analysis

The data were analyzed statistically *via* one-way ANOVA tests followed by Tukey tests. The software package SPSS v22.0 was employed (IBM, Chicago, IL, United States). Numerical values are expressed in the form *mean* ± *SD* and *p* < 0.05 was taken to imply statistical difference.

## Results and Discussion

### Molecular Weights, Monosaccharide Composition, and IR Analysis of the FVPs

**Figure 1:**
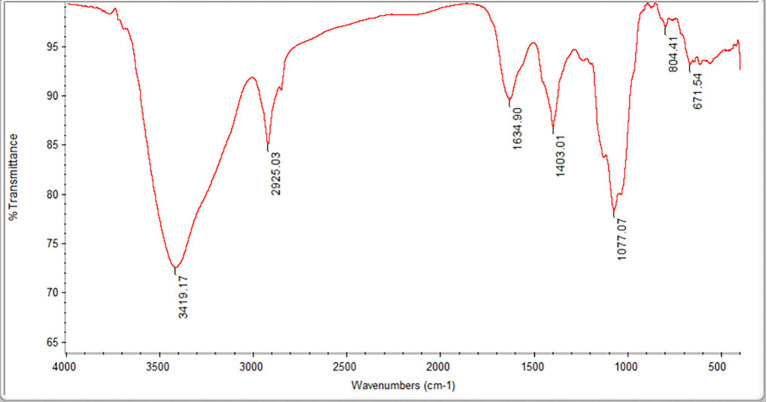
Fourier transform infrared (FT-IR) spectrum of the *Flammulina velutipes* polysaccharides (FVPs).

The *M*_w_ and *M*_n_ values of the FVPs were found to be 2,779,371 and 7,555 g/mol, respectively, and their monosaccharide composition is shown in Table 1. The FT-IR spectrum obtained for the FVPs is shown in Figure 1.

**Table 1:** Monosaccharide composition of the FVPs.

Monosaccharide composition (mg/kg)								
Mannose	Ribose	Rhamnose	Glucuronic acid	Galacturonic acid	Glucose	Galactose	Xylose	Fucose
68796.89	539.35	243.35	50.64	96.24	54900.80	88339.75	22228.41	30248.37

The large IR absorption peak at 3,419 cm^−1^ suggests the possible presence of double bonds, amidogen, and phenyl rings. The band at 2,925 cm^−1^ is probably due to –CH2 antisymmetric stretching vibrations. The presence of carbonyl groups is suggested by the peaks at 3,419 and 1,634 cm^−1^. The strong absorption at 3,100–3,500 and 1,403 cm^−1^ indicates the presence of amidogen. The bands around 1,077 cm^−1^ indicate the possible existence of ether groups.

### Amelioration of the Clinical Symptoms of CCl_4_-Induced Liver Injury

A number of free radicals can be generated from CCl_4_ including trichloromethyl which is produced when CCl_4_ is metabolized by cytochrome P450 (He et al., 2016). The trichloromethyl radical and oxygen work together to damage microsomal lipids in the liver and phospholipid molecules in hepatocyte membranes, initiating lipid peroxidation (Guo et al., 2015). The damage caused to the structure of the hepatic cellular membranes increases their permeability allowing ALT and AST to infiltrate into the blood. In addition, large amounts of TG and TC are deposited in the hepatocytes, leading to an increase in the TG and TC content of the serum (Lykkesfeldt, 2007).

**Figure 2:**
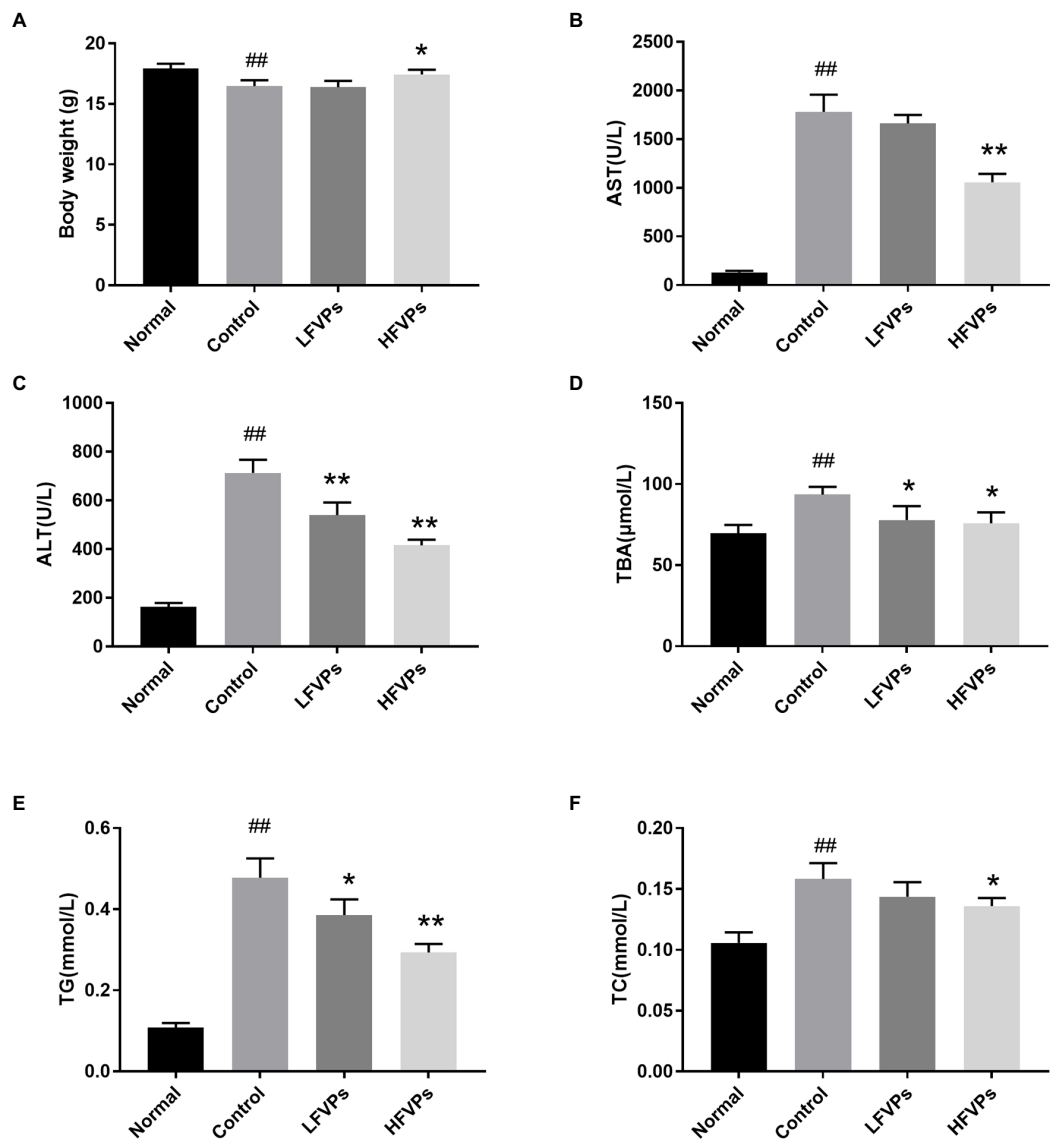
Effect of treatment with FVPs on **(A)** body weight and content of certain biochemicals in the serum: **(B)** aspartate transaminase (AST), **(C)** alanine aminotransferase (ALT), **(D)** total bile acid (TBA), **(E)** triglyceride (TG), and **(F)** total cholesterol (TC). The data are presented in the form: *mean* ± *SD* (with eight mice per group). The symbols # and ## represent significance levels of *p* < 0.05 and *p* < 0.01 compared to the Normal group, respectively. Similarly, * and ** represent *p* < 0.05 and *p* < 0.01 compared to the Control group, respectively.

Results of biochemical assays are presented in Figure 2. We first note that the average body weight of the mice whose livers had been injured by CCl_4_ was significantly less than that of the healthy mice (Figure 2A). However, the body masses of those treated with FVPs (especially those in the HFVPs group) were significantly improved (*p* < 0.05) due to their improved physical condition.

The AST, ALT, TG, TC, and TBA contents of the serum are all significantly increased due to CCl_4_ intoxication (compared to healthy mice) which are showed in Figure 2. However, pretreatment with the FVPs significantly reduced these elevated serum levels in a dose-dependent manner. That is, the higher dose group (200 mg/kg BW) experienced stronger hepatoprotective effects.

**Figure 3:**
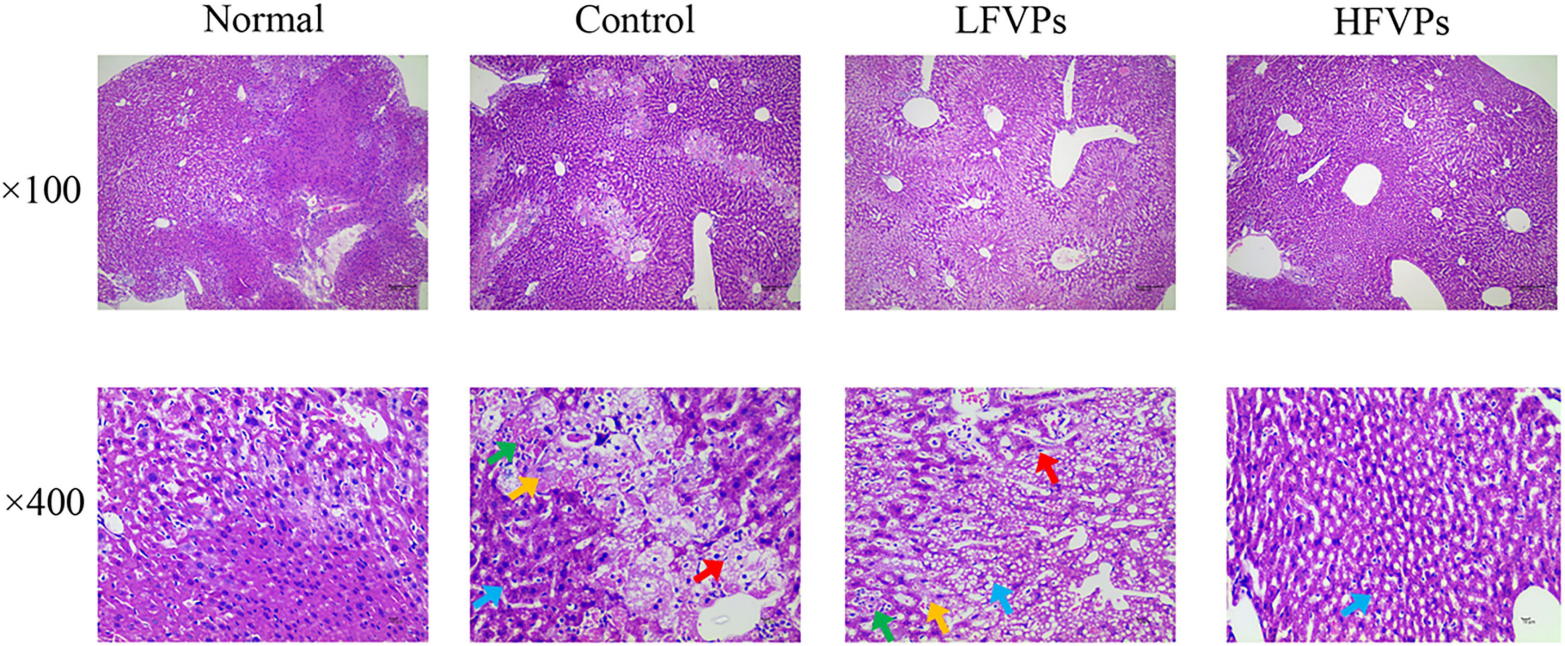
Effect of FVPs treatment on liver morphology. (HE-stained tissue images at magnifications of 100× and 400×) Yellow arrow: edema and degeneration of hepatic cells. Blue arrow: Hepatocyte steatosis. Green arrow: necrosis of liver cells. Red arrow: proliferation of fibroblasts.

The histopathological changes that occurred in the liver tissues of the mice are presented in Figure 3 and Supplementary Table 2. In the Normal group, the liver cells are arranged in an orderly manner from the central vein and their nuclei are prominent and cytoplasm uniform. Treatment with CCl_4_ can be seen to induce extensive liver damage. The damage is characterized by inflammatory cell infiltration and serious cellular degeneration. However, the state of the malignancy is strongly ameliorated in the HFVPs group as shown by the reduction in the number of large vacuoles formed and extent of the inflammatory infiltration and cellular degeneration.

### Effect of FVPs on Hepatic Antioxidant Activity

Antioxidant enzymes such as SOD and CAT play important roles in scavenging ROS. SOD first catalyzes the conversion of O_2_^−^ to H_2_O_2_ and then CAT scavenges the H_2_O_2_ to form O_2_ and H_2_O, thus inhibiting lipid peroxidation (Miranda et al., 2000; Valko et al., 2007). When lipid peroxidation does occur, the terminal product is MDA. Hence, the MDA level can be used to assess the extent of the injury caused by peroxidation (Abuja and Albertini, 2001). Similarly, PC is the oxidative product of amino acid side chains and can be used as an effective indicator to assess the degree of protein oxidative injury (Dalle-Donne et al., 2003).

**Figure 4:**
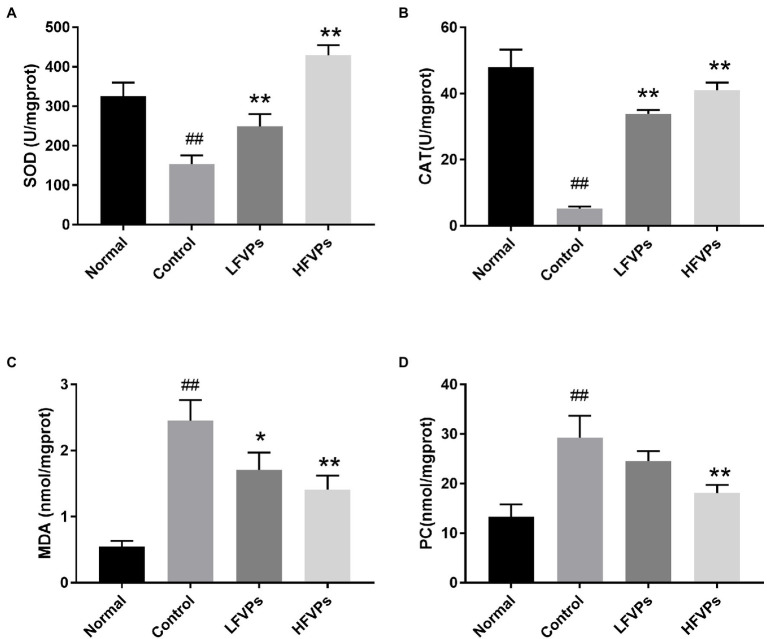
Effect of FVPs on hepatic antioxidant activity. The bar charts show the results obtained for the hepatic content of **(A)** superoxide dismutase (SOD), **(B)** catalase (CAT), **(C)** malonaldehyde (MDA), and **(D)** protein carbonyl (PC; as measured using ELISA kits). The data are presented in the form: *mean* ± *SD* (with eight mice per group). The symbols # and ## represent significance levels of *p* < 0.05 and *p* < 0.01 compared to the Normal group, respectively. Similarly, * and ** represent *p* < 0.05 and *p* < 0.01 compared to the Control group, respectively.

The results obtained for the four indicators mentioned above are showed in Figure 4. As can be seen, the administration of CCl_4_ significantly decreases the activity of SOD and CAT and leads to the accumulation of MDA and PC in the liver (*p* < 0.01). However, pretreatment with FVPs resulted in a notable elevation of SOD and CAT activity and reduction in the amount of MDA and PC produced among the CCl_4_-treated groups. Thus, the FVPs are able to attenuate the liver damage induced by CCl_4_ administration. In addition, the higher dose of FVPs manifested a better anti-oxidant ability (*p* < 0.01).

### Effect of FVPs on Hepatic Inflammation

The damage caused by oxidative stress leads to the release of inflammatory mediators, e.g., IL-1β, IL-6, and TNF-α (Khan et al., 2019). MPO activity can also be used to indicate the degree of inflammation as it serves as a marker to evaluate the level of neutrophilic infiltration (Chen et al., 2020).

**Figure 5:**
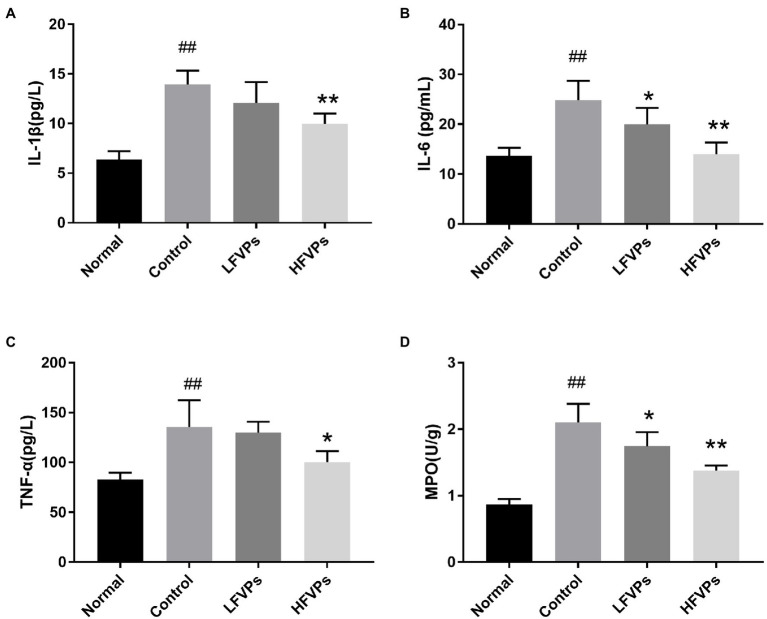
Effect of FVPs on hepatic inflammation. The bar charts show the results obtained for the hepatic content of **(A)** IL-1β, **(B)** IL-6, **(C)** TNF-α, and **(D)** myeloperoxidase (MPO; as measured using ELISA kits). The data are presented in the form: *mean* ± *SD* (with eight mice per group). The symbols # and ## represent significance levels of *p* < 0.05 and *p* < 0.01 compared to the Normal group, respectively. Similarly, * and ** represent *p* < 0.05 and *p* < 0.01 compared to the Control group, respectively.

The IL-6, IL-1β, TNF-α, and MPO contents were all increased after CCl4 was administered to the Control group (*p* < 0.01) which were showed in Figure 5. Furthermore, treatment with FVPs markedly weakened the production of the pro-inflammatory cytokines (IL-6, IL-1β, and TNF-α) in a dosed manner, as was the MPO level. The higher dose of FVPs (200 mg/kg BW) substantially enhanced the anti-inflammatory effect.

### Gut Microbiota Analysis

**Figure 6:**
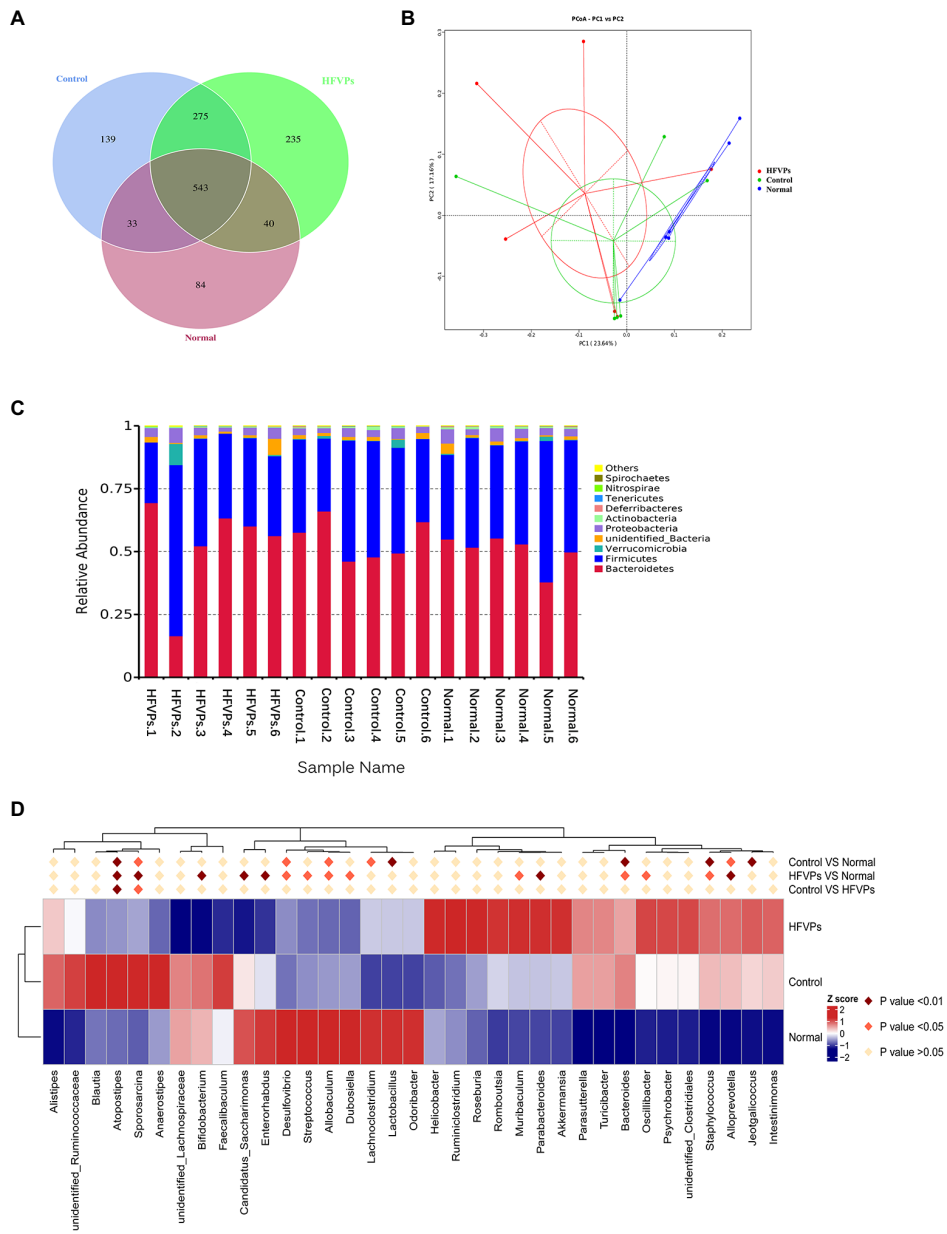
**(A)** Venn diagram showing the number of operational taxonomic units (OTUs) in the Normal, Control, and HFVPs groups. **(B)** Principle coordinate analysis (PCA) plot used to evaluate the beta diversity parameters. Each point represents an individual specimen. **(C)** Microbial composition of the Normal, Control, and HFVPs groups at the genus level (top 20). **(D)** Heatmap of the significant differences in the gut microbiomes in the Normal, Control, and HFVPs groups at the genus level (obtained using the Metastats method). The data are presented in the form *mean* ± *SD* (*n* = 6).

More and more studies have shown that the gut microbiota is involved in liver disease pathogenesis as the composition of the gut microbiota will significantly influence the nature of the gut-derived products that are leaked (Krishnan et al., 2018; Yip et al., 2018). To investigate whether changing the gut microbiota can facilitate any of the metabolic improvements involved in the treatment of mice with FVPs, the intestinal contents of the mice were collected and analyzed to reveal the nature of their gut microbiota. The results are shown in Figure 6.

The mice in the Normal, Control, and HFVPs groups share 543 OTUs which are showed in Figure 6A. Treatment with FVPs led to 235 unique OTUs appearing in the gut microbiota of the treated mice.

The Shannon and Simpson indices can be used to evaluate the diversity of the bacterial communities and the ACE and Chao1 indices can be used to gauge their richness. The values of these indices calculated for three of the experimental groups of mice are showed in Table 2. The table implies that the high-dose FVPs treatment had little effect on the alpha diversity of the gut microbiota compared with Control group. Principle coordinate analysis (PCA) plot to evaluate the beta diversity parameters of gut microbiota is showed in Figure 6B. The specimens of three groups did not separate completely reflecting they shared some of same composition of gut microbiota.

**Table 2:** Alpha diversity evaluated using the ACE, Chao1, Simpson, and Shannon indices.

Index	Treatment		
	Normal	Control	HFVPs
Shannon	5.48 ± 0.21	5.89 ± 0.34^#^	5.56 ± 0.94
Simpson	0.94 ± 0.00	0.96 ± 0.01	0.93 ± 0.06
Chao1	546.70 ± 21.48	599.41 ± 172.12	635.70 ± 132.54
ACE	513.34 ± 13.88	571.56 ± 154.84	604.82 ± 122.65

The data are expressed in the form mean ± *SD* (*n* = 6).

# symbol represents a statistical difference compared to the Normal group at the *p* < 0.05 level.

The microbial composition in the Normal, Control, and HFVPs groups at the phylum level is illustrated in Figure 6C. In agreement with published studies, the species dominating the gut microbiota in this study are *Bacteroidetes* and *Firmicutes*. These species matter as they play a role in the body’s energy-balance mechanism as they affect energy transformation, nutrient absorption, and glucose metabolism (Turnbaugh et al., 2006). The Firmicutes/Bacteroidetes ratio is significantly reduced to 0.7462 ± 0.2439 after CCl_4_ intoxication, while Normal group is 0.8792 ± 0.3129. But this ratio is raised to 1.1602 ± 1.4553 in the groups treated with HFVPs (Supplementary Table 3). This implies that the FVPs may have the ability to raise the number of calories absorbed from the food by changing the composition of the gut microbiota.

Significantly different gut microbiomes in the Normal, Control, and HFVPs groups at the genus level is revealed in Figure 6D. *Lactobacillus* has been reported to have a hepatoprotective effect by inhibiting β-glucuronidase productivity of the intestinal microflora (Han et al., 2005). The abundance of *Lactobacillus* is significantly reduced after treatment with CCl_4_ (compared to the Normal group). However, the administration of FVPs elevates the abundance of *Lactobacillus* in the gut. The increment in *Ruminococcus* could independently indicate the occurrence of significant liver fibrosis (Boursier et al., 2016). According to the heatmap in Figure 6D, *Ruminococcaceae* shows increase in the Control group compared with the Normal group. However, the FVPs lower the abundance of *Ruminococcaceae*. An increase in *Bacteroides* suggests the potential occurrence of nonalcoholic fatty liver disease (Meng et al., 2018). This applies to the Control group, but the FVPs substantially help mitigate the situation according to Figure 6D. We also found that the abundance of *Atopostipes* and *Sporosarcina* are both significantly different in the two-by-two comparison of Normal, Control, and HFVPs groups. CCl_4_ treatment elevated the abundance of *Atopostipes* and *Sporosarcina* in gut, and HFVPs effectively reduced them. But the role of *Atopostipes* and *Sporosarcina* in gut is rarely studied, especially in the gut microbiota involved in liver disease. *Atopostipes* and *Sporosarcina* perhaps will be the biomarker for hepatoprotective gut microorganisms, which still need further study.

### Metabolite Analysis

Gut microbiota mediate metabolic activity by digesting various dietary compounds and supplying micronutrients. On the other hand, dietary compounds also influence the growth and metabolic activity of gut microbiota and therefore have potential health effects (Farag et al., 2020).

**Figure 7:**
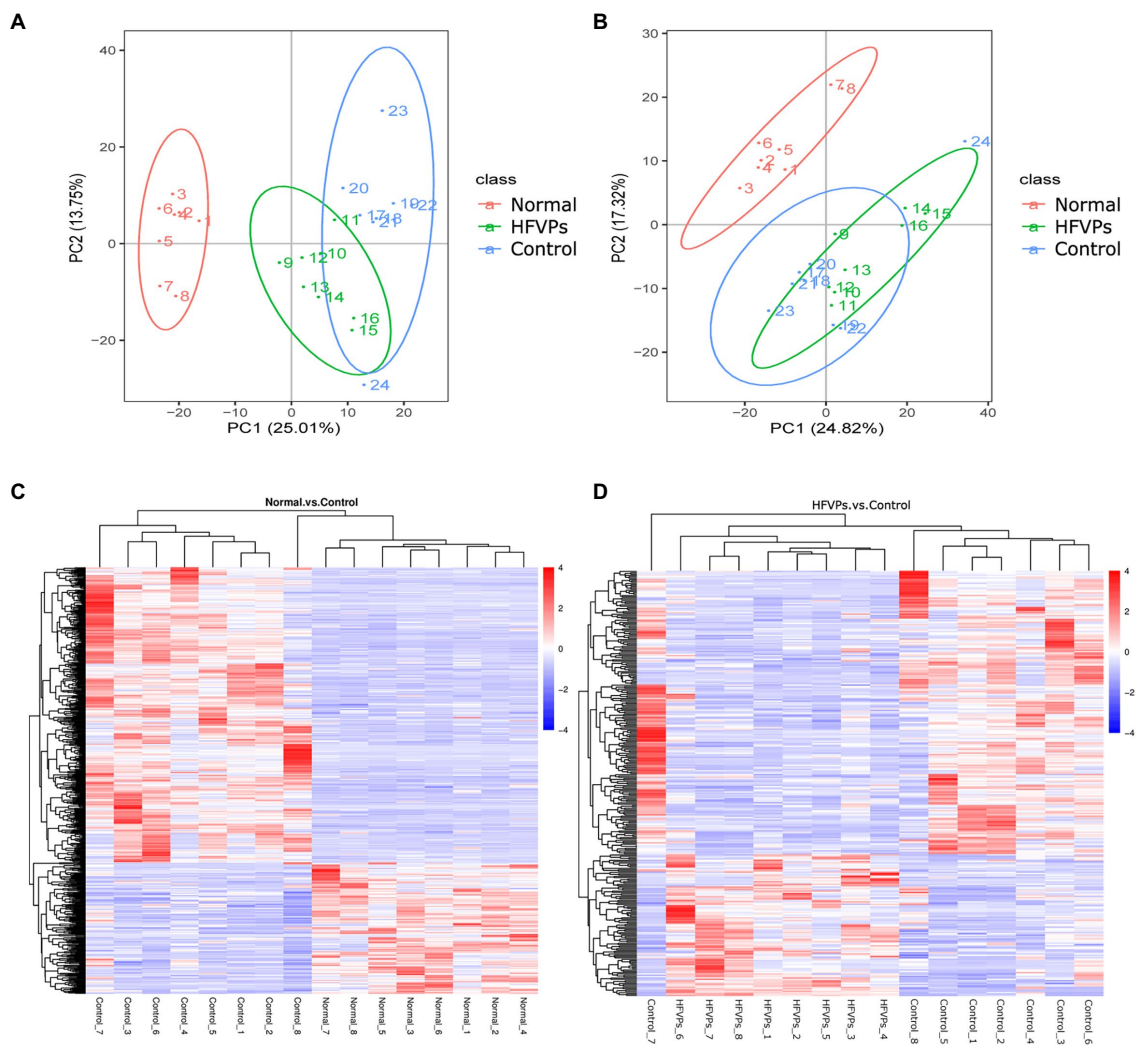
**(A)** Principle coordinate analysis plots of the fecal metabolites in the Control, Normal, and HFVPs groups in positive mode. **(B)** PCA plots of the fecal metabolites in the Control, Normal, and HFVPs groups in negative mode. **(C)** Hierarchical clustering plots of the significantly changed metabolites in the Control and Normal groups. **(D)** Hierarchical clustering plots of the significantly changed metabolites in the Control and HFVPs groups.

Clear separations between the Normal and Control groups are showed in Figures 7A,B. This implies that treatment with the toxin CCl_4_ changes the normal gut microbiota metabolites in mice. There was also some changes in PCA plots between Control and HFVPs group, reflecting HFVPs had effects on gut microbiota metabolites in liver injury mice. In total, 1,793 metabolites were detected and the heatmaps show they are independent and unique in the metabolome dimensions (Figures 7C,D). Between the Normal and Control groups, 182 metabolites were upregulated and 412 were downregulated. In the HFVPs and Control groups, 106 metabolites were upregulated and 212 were downregulated. Supplementary Table 1 gives further information on the 318 metabolites that changed significantly in the HFVPs group compared with the Control group.

**Figure 8:**
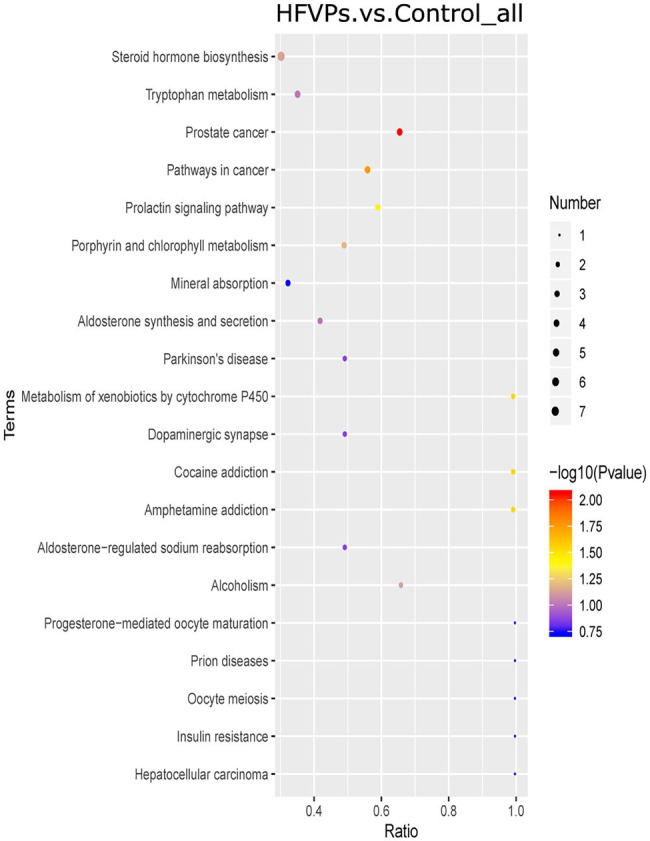
Kyoto Encyclopedia of Genes and Genomes (KEGG) pathway enrichment plot of the significantly changed metabolites in the Control and HFVPs groups.

The KEGG database was employed to analyze the top 20 enriched pathways related to the significantly different metabolic changes between the Control and HFVPs groups, giving the results shown in Figure 8. As can be seen, the most enriched pathways include those related to steroid hormone biosynthesis, tryptophan metabolism, cancer pathways, xenobiotics metabolism by cytochrome P450, aldosterone synthesis and secretion, and insulin resistance.

The induction of fatty acid biosynthesis and transportation leads to intrahepatic lipid accumulation, which contributes to the development of nonalcoholic fatty liver disease (Buzzetti et al., 2016). Myristic acid (Com_17487_neg) and stearic acid (Com_38_neg) are saturated fatty acids that are enriched in the fatty acid biosynthesis pathway. These are downregulated in the HFVPs group, which helps improve liver function. Xanthurenic acid (Com_11899_pos), indole (Com_1868_pos), and kynurenic acid (Com_5832_neg) are tryptophan-derived bacterial metabolites enriched in the tryptophan metabolism pathway. It has been found that indole, and other tryptophan-derived bacterial metabolites, attenuate the expression of pro-inflammatory cytokines in macrophages and also modulate the inflammatory response in hepatocytes (Krishnan et al., 2018). The downregulation of xanthurenic acid (Com_11899_pos), indole (Com_1868_pos), L-Tryptophan (Com_185_pos), and kynurenic acid (Com_5832_neg) in the HFVPs group may result from the more consumption for anti-inflammatory effect, such as inhibition release of IL-6, IL-1β, and TNF-α in liver.

The metabolites aflatoxin M1 (Com_14447_pos) and aflatoxin B1 (Com_23988_pos) that are enriched in the metabolism of xenobiotics by cytochrome P450 pathway are downregulated in the HFVPs group. As the CCl_4_ is biotransformed to ROS *via* the hepatic cytochrome P450 system, the lower level of the metabolism of xenobiotics by cytochrome P450 pathway may contribute to the hepatoprotective effect of the FVPs (Qi et al., 2014).

Overall, the bacterial metabolites in the HFVPs group might protect the liver from the effects of CCl_4_ intoxication *via* the fatty acid biosynthesis, tryptophan metabolism, and xenobiotic metabolism by cytochrome P450 pathways, etc.

## Conclusion

The results show that treatment with FVPs attenuates the level of hepatic injury by promoting antioxidant and anti-inflammatory effects. Furthermore, our analysis of the gut microbiota and bacterial metabolites illustrates that the FVPs change the composition of the gut microbiome and regulate certain bacterial pathways associated with fatty acid biosynthesis, tryptophan metabolism, and metabolism of xenobiotics by cytochrome P450 to protect the liver from the toxic effects of CCl_4_. Our study thus provides a deeper understanding of the hepatoprotective effects and modulation of bacterial metabolites brought about by treatment with FVPs.

## Data Availability Statement

The datasets presented in this study can be found in online repositories. The names of the repository/repositories and accession number(s) can be found at: https://www.ncbi.nlm.nih.gov/, PRJNA793715.

## Ethics Statement

The animal study was reviewed and approved by the Animal Care and Use Committee of the Sichuan Provincial People’s Hospital. Written informed consent was obtained from the owners for the participation of their animals in this study.

## Author Contributions

YW conceived the article. YX wrote this manuscript. ZZ, BW, XH, JT, WP, and JZ contributed in carrying out experiments and data analysis. All authors contributed to the article and approved the submitted version.

## Funding

This study was supported by the National Modern Agro-industry Technology Research System (CARS-24), Sichuan Edible Mushroom Innovation Team and Local Financial Funds of National Agricultural Sciences and Technology Center, Chengdu (grant no. NASC2020AR06).

## Conflict of Interest

The authors declare that the research was conducted in the absence of any commercial or financial relationships that could be construed as a potential conflict of interest.

## Publisher’s Note

All claims expressed in this article are solely those of the authors and do not necessarily represent those of their affiliated organizations, or those of the publisher, the editors and the reviewers. Any product that may be evaluated in this article, or claim that may be made by its manufacturer, is not guaranteed or endorsed by the publisher.

## Footnotes

^1^ https://www.arb-silva.de/

^2^ http://www.lipidmaps.org/

^3^ http://www.hmdb.ca/

^4^ http://www.genome.jp/kegg/
